# SAUSI: a novel assay for measuring social anxiety and motivation

**DOI:** 10.1101/2024.05.13.594023

**Published:** 2024-05-14

**Authors:** Jordan Grammer, Rene Valles, Alexis Bowles, Moriel Zelikowsky

**Affiliations:** Department of Neurobiology, University of Utah, United States

## Abstract

Social anxiety is one of the most prevalent mental health disorders, though the underlying neurobiology is poorly understood. Progress in understanding the etiology of social anxiety has been hindered by the lack of comprehensive tools to assess social anxiety in model systems. Here, we created a new behavioral task – Selective Access to Unrestricted Social Interaction (SAUSI), which combines elements of social motivation, hesitancy, decision-making, and free interaction to enable the wholistic assessment of social anxiety-like behaviors in mice. Using this novel assay, we found that social isolation-induced social anxiety-like behaviors in female mice are largely driven by increases in social fear, social hesitancy, and altered ultrasonic vocalizations. Deep learning analyses were able to computationally identify a unique behavioral footprint underlying the state produced by social isolation, demonstrating the compatibility of modern computational approaches with SAUSI. Finally, we compared the results of SAUSI to traditionally social assays including the 3-chamber sociability assay and the resident intruder task. This revealed that behavioral changes induced by isolation were highly context dependent, and that while fragments of social anxiety measured in SAUSI were replicable across other tasks, a wholistic assessment was not obtainable from these alternative assays. Our findings debut a novel task for the behavioral toolbox – one which overcomes limitations of previous assays, allowing for both social choice as well as free interaction, and offers a new approach for assessing social anxiety in rodents.

## INTRODUCTION

Social isolation causes a myriad of physiological, emotional, and behavioral deficits across species (Cacioppo et al., 2014; Grammer & Zelikowsky, 2022; National Academies of Sciences, Engineering, and Medicine, 2020; Zelikowsky et al., 2018a). One of the most detrimental impacts of social isolation is its ability to promote social anxiety (Eres et al., 2023; Lim et al., 2016; Teo et al., 2013). Despite the negative impact of social isolation to engender social anxiety, it has been difficult to make progress in our understanding of the neurobiology underpinning this state, as complex behavioral assays to study social anxiety-like behavior in model systems has been largely lacking (Carlton et al., 2020; Mathew et al., 2001; Réus et al., 2014; Toth & Neumann, 2013). Indeed, the majority of social anxiety experiments conducted in rodents have used social interaction – or a lack thereof – as an indirect index of social anxiety, often measured by time spent in proximity to a conspecific restricted behind a barrier (Litvin et al., 2013; Toth & Neumann, 2013). Alternative assays have been developed to examine social motivation – or the degree to which an animal is willing to “work” for access to a conspecific, as an index of social reward or social aversion (Bannai et al., 2007; Dölen et al., 2013; Falkner et al., 2016; Fish et al., 2002, 2005; Hu et al., 2021; Lee et al., 2024; Maloney et al., 2023; Martin & Iceberg, 2015). Others have observed interaction and vigilance in the presence of a conspecific (Luo et al., 2022; Steinman et al., 2019) or free interaction behaviors including social investigation, avoidance, and aggression (Gómez et al., 2017) as indirect measurements of social anxiety. While many intricate paradigms have been implemented to generate social anxiety in rodent models (Carlton et al., 2020; Toth et al., 2012; Toth & Neumann, 2013), there has be limited progress in novel tasks for assessment of social anxiety. Such work has largely utilized two types of tasks: a “free interaction” assay, such as the resident intruder assay, which allows for assessment of natural interaction between two agents, but lacks the ability to probe social motivation or choice. Or, a “social motivation” assay, such as the three-chamber assay, which allows for identification of motivated behavior, but which fails to capture naturalistic, interactive behavior between agents, as stimulus animals are often restrained or contained in some way (Kemble, 1993; Koolhaas et al., 2013a; Nadler et al., 2004; Toth & Neumann, 2013; Yang et al., 2011a). Thus, researchers are able to examine natural social interaction *or* social motivation, but not both at the same time. Given that social anxiety is comprised of changes to both social motivation as well as alterations in naturalistic interactions (Administration, 2016; Rose & Tadi, 2024; Toth & Neumann, 2013), current assays fail to provide a way in which to comprehensively examine social anxiety-like behavior. Here, we address this gap in our behavioral toolkit and introduce a novel assay, Selective Access to Unrestricted Social Interaction (SAUSI), which allows for examination of *both* social motivation and free social interaction within a single assay, thereby enabling us to comprehensively examine social anxiety-like behavior.

Recent years have seen an explosion of computational tools and analysis pipelines for the estimation and analysis of complex animal behavior. For example, Social Leap Estimates Animal Poses (SLEAP) (Pereira et al., 2022), DeepLabCut (A. Mathis et al., 2018), Mouse Action Recognition System (MARS) (Segalin et al., 2021) are programs which utilize deep learning algorithms to track body points of multiple mice with identical features. Large datasets generated by postural tracking software are then used for further supervised and/or unsupervised analysis approaches. Supervised behavioral analysis programs such as Mouse Action Recognition System (MARS) (Segalin et al., 2021) and Simple Behavioral Analysis (SimBA) (Nilsson et al., 2020) allow behaviors identified by an experimenter to be automatically scored, reducing user error, bias, and differences in scoring across and even within labs (Nilsson et al., 2020; Segalin et al., 2021). Unsupervised behavioral analysis pipelines such as Motion Mapper (Berman et al., 2014) and MoSeq (Wiltschko et al., 2015) can computationally identify patterns of motion, detecting clusters comprised of behaviors, poses, and movements that could not otherwise be detected with the human eye. More machine learning programs for behavior analysis include Stytra (Štih et al., 2019), Anipose (Karashchuk et al., 2021), Janelia Automatic Animal Behavior Annotator (JAABA) (Kabra et al., 2013), DeepEthogram (Bohnslav et al., 2021), TRex (Walter & Couzin, 2021), Ctrax (Branson et al., 2009), OptiMouse (Ben-Shaul, 2017), DeepPoseKit (Graving et al., 2019), 3-Dimensional Aligned Neural Network for Computational Ethology (DANNCE) (Dunn et al., 2021), and 3D virtual mouse (Bolaños et al., 2021), highlighting the speed at which innovation in this space is occurring. Beyond behavior, interfaces such as DeepSqueak (Coffey et al., 2019), VocalMat (Fonseca et al., 2021), Mouse Ultrasonic Profile ExTraction (MUPET) (M et al., 2017), HybridMouse (Goussha et al., 2022), and Joseph the MoUSE – Mouse Ultrasonic Sound Explorer (Kania et al., 2024) have revolutionized analysis of ultrasonic vocalizations through automated detection, segmentation and deep learning analyses. These tools not only reduce bias and increase efficiency, but also allow us to visualize behavioral and vocal changes in innovative and novel ways. Thus, the development of novel behavioral assays which lend themselves to these techniques is pivotal in a new age of validity, reproducibility, and translational meaning (Ghosh et al., 2024).

Here, we developed SAUSI, which allows for assessment of motivated social choice, hesitancy, decision making, and free social interaction between pairs of mice, and which lends itself to modern machine learning and computational approaches. Next, we tested the impact of chronic social isolation to produce social anxiety-like behavior using SAUSI. We found that social isolation promotes a state of social anxiety, characterized by multiple factors including social freezing, social hesitancy, and changes in ultrasonic vocalizations, but did not alter prosocial interaction or social motivation. We demonstrate the ability to apply computational techniques to SAUSI-generated behavioral data, and identify isolation state-specific behavioral motifs. Critically, we find these effects to be sex-specific, with social anxiety-like behavior characteristic of females but not males. Finally, we demonstrate that SAUSI uniquely reveals social anxiety-like behaviors when compared to traditional assays conducted in the home cage or in a three-chamber assay. These experiments highlight SAUSI as a novel assay for a modern neuroscience toolkit, well equipped for the behavioral and computational analysis of complex social behaviors such as social anxiety.

## RESULTS

### Selective Access to Unrestricted Social Interaction (SAUSI): a novel assay to assess social motivation and social anxiety.

**Figure 1.**
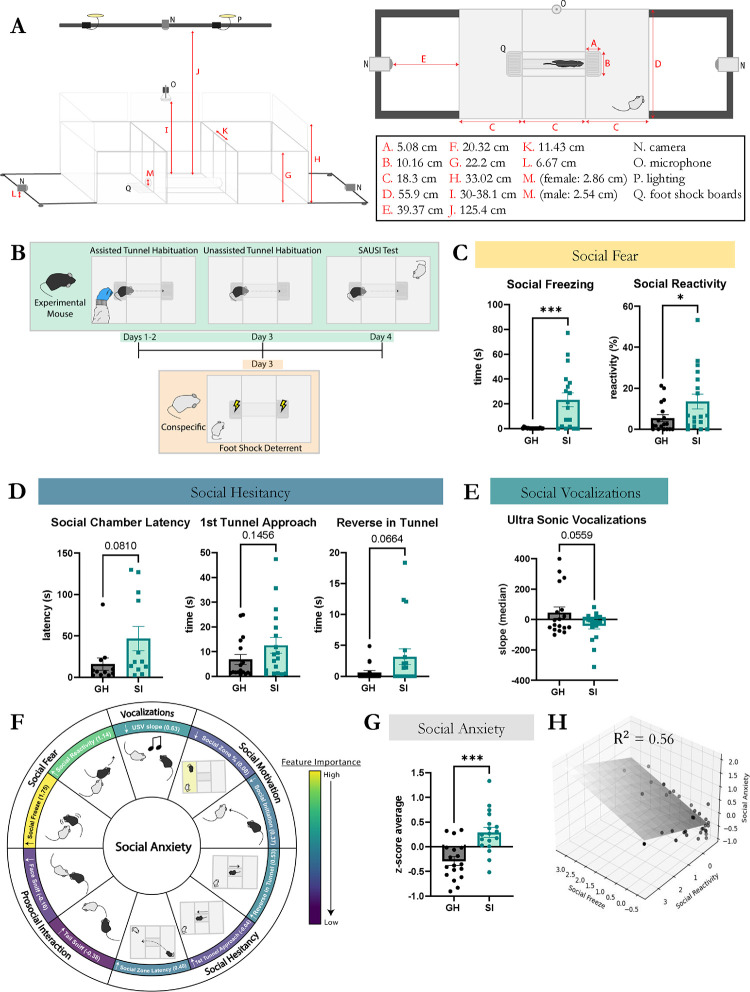
SAUSI – a novel assay to probe social anxiety. (**A**) SAUSI apparatus side view (left panel) and SAUSI apparatus top view (right panel). Numbers indicate the measurements (in centimeters) for each labelled arrow in the diagrams. (**B**) SAUSI protocol. Experimental mice are habituated to the tube for three days (green). Conspecifics receive one day of tube deterrent training (yellow). (**C**) Social fear was significantly increased by isolation. (**D**) Social hesitancy was increased by isolation. (**E**) Social vocalizations negatively altered by isolation. (**F**) Summary of SAUSI behaviors that combine to generate social anxiety. Logistic regression was performed to determine which factors are most influential to the social anxiety score. Feature importance is color coded, with the logistic regression coefficients labelled. Arrows indicate the direction of SI mice relative to GH mice. (**G**) Social Anxiety represented as the z-score average of all behaviors in panel C. (**H**) Largest contributions to the social anxiety score are social fear behaviors. Multi-linear regression showing strong correlation between social fear behaviors and the social anxiety score. (n=18, independent samples t-tests)

A number of mental health disorders are known to include elements of social anxiety, yet we lack the ability to adequately identify social anxiety in model systems, limiting the ability to interrogate the neurobiology underlying this state (Réus et al., 2014). To begin to address this, we developed a novel behavioral assay, Selective Access to Unrestricted Social Interaction (SAUSI), to allow for the wholistic assessment of social anxiety-like behaviors. Key to our design was the development of a task that would allow us to probe social motivation as well as free social interaction. As a first step, we designed a novel arena which would allow us to combine free, naturalistic interactions between an experimental mouse and a conspecific mouse in an open space (“social chamber”), free behavior of the experimental mouse in a distinct, independent space (“home” chamber), and motivated social behavior, wherein the experimental mouse can choose to access a conspecific or withdraw from interaction via a tunnel that connects the two chambers (Fig. 1a–b). Similar to the 3-chamber task for sociability, the two outer chambers allow us to measure social motivation factors, such as how much time a mouse prefers to spend in the social vs. home chamber. However, instead of limiting social interaction by physically restricting the conspecific under a barrier, SAUSI allows for free interaction between the two mice when in the social chamber. Conspecifics are prevented from crossing to the opposite “home” side by receiving five minutes of footshock deterrent training prior to the social test (Fig. 1a–b). We chose to connect the two outer chambers with a narrow tunnel, which is compatible with hierarchy testing (Fan et al., 2019). This provides physical segregation between the two chambers, further preventing unwanted crossing by the conspecific. The tunnel also allows for measurement of precise time points in the target mouse’s social decision making as they approach the social chamber, and reveals hesitancy in mice when they shelter in the tunnel for discrete periods of time or decide to back out after initiating a crossover.

Footshock boards were designed as the primary form of deterrent to keep conspecifics from crossing to the opposite side (Macheda et al., 2020; Modrak, 2023). BALB/c mice were used as conspecifics, as this strain has been shown to be relatively docile, removing potential confounds related to intruder aggression (Potter, 2012). To ensure that conspecific mice do not cross over into the “home” chamber or block the tunnel entryway, we employed an entry deterrent strategy, wherein a mild footshock was delivered via an electric board directly in front of the tunnel (Fig. 1a). Footshock boards were connected to grid scramblers (Med Associates), with mice completing the loop if at least one limb touched the positive and one limb touched the negative components of the footshock board (Figure 1 – figure supplement 1). To determine the bare minimum shock intensity (mA) required to serve as a deterrent without significantly altering the conspecifics’ behavior, we conducted a behavior response curve experiment (Figure 1 – figure supplement 2a). Female BALB/c mice were shocked at either .1mA., .2mA, .3mA or .5mA whenever they touched the footshock board across a 5-minute training session. Mice were given 1 session/day for 2 days and then tested with a group housed female C57BL/6 mouse on the third day to assess social behavior and efficacy of the foot shock training (Figure 1 – figure supplement 2a) . We found that .5 and .3 mA shocks, but not .1mA or .2mA, were sufficient to act as deterrents by significantly reducing the amount of time and latency of stepping on the foot shock board (Figure 1 – figure supplement 2b). However, .5mA resulted in significant changes to conspecific behavior, such as increased freezing (Figure 1 – figure supplement 2c). In contrast, .3mA worked as a deterrent while not significantly impacting conspecific behavior (Figure 1 – figure supplement 2c). There were no changes in sniffing, thigmotaxis, or aggression across all groups. Locomotion was reduced by all levels of shock, even those that weren’t effective as a deterrent (Figure 1 – figure supplement 2c). Critically, .3mA shock resulted in no changes to behavior of the experimental mouse when interacting with the mildly shocked conspecific Figure 1 – figure supplement 2d).

Many neuroscience experiments rely on tethering and head mounting during behavior to understand neural dynamics in real time. Because of this we also adapted SAUSI to accommodate head mounts and tethering by piloting an open-top tunnel design. This features a U-shaped channel in between the two outer chambers instead of an enclosed tunnel. This has been shown to work with miniature microscopes (Inscopix) as well as tethering for optogenetics experiments (Supplemental Video 1).

Collectively, this design allows for the assessment of a) free social interaction, b) solitary behaviors, c) social motivation, and d) social hesitancy and social decision making. All of these features combined enable a robust paradigm for assessing social anxiety-like behaviors.

### Prolonged social isolation promotes social anxiety, as revealed by SAUSI.

Social isolation (SI) is known to promote social anxiety (Eres et al., 2023; Lim et al., 2016; Teo et al., 2013). Yet, to date, we have been unable to consistently test this in rodent models due to the lack of an appropriate, wholistic assay that can measure multiple features of social anxiety-like behavior (Réus et al., 2014; Toth & Neumann, 2013). We developed SAUSI to test whether prolonged SI results in increased social anxiety-like behavior or reduced social motivation in mice. We first tested females, as they are known to exhibit significantly greater levels of social anxiety compared to males (Asher et al., 2017; Asher & Aderka, 2018). We found that prolonged social isolation produced an increase in social fear behaviors such as social freezing (freezing in response to being sniffed by the conspecific) and social reactivity (jumping or darting in response to sniffing) (Fig. 1c) (Bauer & Gariépy, 2001; Gruene et al., 2015; Trott et al., 2022). These two behaviors were categorized as social fear based on previous literature discussing features of social fear in rodents (Toth & Neumann, 2013). Isolation also increased social hesitancy behaviors such as latency to approach the social chamber, more time spent sheltering in the tunnel upon the first approach, and reversing out of the tunnel once the decision was made to cross over to the social chamber (Fig. 1d). Finally, isolation decreased the slope of ultrasonic vocalizations (USVs), indicating changes in mouse vocal communication (Fig. 1e). Interestingly, while we hypothesized that there would be a decrease in social motivation and alterations prosocial behaviors (social preference, prosocial initiation, and sniffing behaviors), we only found a reduction in social initiation in SI mice compared to GH (Figure 1 – figure supplement 3a–b).

Next, we sought to visualize the behavioral changes observed as a wholistic, cumulative social anxiety-like behavior score. We converted the datasets for each behavior into z-scores and found the average z-score across all behaviors used to index social anxiety (Fig. 1f). This social anxiety z-score was significantly higher in socially isolated mice compared to group-housed (GH) controls (Fig. 1g). We then performed multi-logistic regression analyses to determine if the generated equation could distinguish between GH and SI mice. It was able to do so with a 93% training accuracy. The coefficients of this equation reveal the behavioral features that most heavily mediate our social anxiety score (Fig. 1f). The components which influenced the social anxiety score most strongly were social freezing and social reactivity, indicating that social fear behaviors are the most important factor in the determination of social anxiety in mice (Fig. 1c). Despite the high importance of the social fear scores, 44% of the variance of the social anxiety score was unexplained by social fear, indicating that other factors were important components as well (Fig. 1h). These included social vocalizations and social hesitancy (Fig. 1d–e). Though the slope of USVs was reduced in SI mice, there were no differences in the total number of USVs produced by either group (Figure 1 – figure supplement 3c). Additional behaviors such as mobility, aggression, and thigmotaxis during the baseline phase were also altered by isolation in SAUSI, but SI did not impact non-social freezing (Figure 1 – figure supplement 3d–f).

### Computational approaches + SAUSI can detect the social state of an animal.

Social behavior in rodents is highly complex and dynamic, continually shifting as animals engage in back-and-forth interactions. Recent advances in machine learning and computational approaches for the analysis of social behavior have enabled a deeper, broader, and less biased approach towards understanding social behavior, including the ability to perform automated behavior classification and to identify behavioral patterns beyond what can be determined by the naked eye (Datta et al., 2019; Flavell et al., 2022; Ghosh et al., 2024; Kennedy, 2022; M. W. Mathis & Mathis, 2020; Pereira et al., 2020). To test whether SAUSI lends itself to modern computational approaches for postural tracking and analysis of behavior, we applied a variety of computational tools to videos obtained from our SAUSI assay (behavior displayed in Fig. 1).

**Figure 2.**
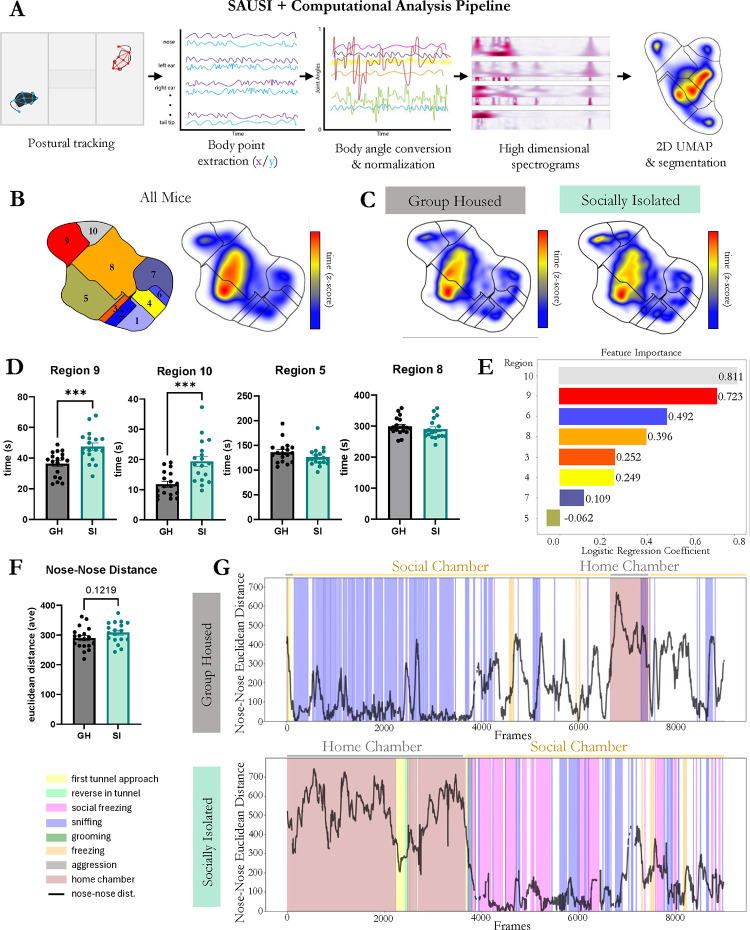
Examining the effects of SI using computational methods. (**A**) The computational workflow for unsupervised behavioral classification. (**B**) Map of behaviors that were generated by the Motion Mapper pipeline. (**C**) Heatmap distribution of time on the behavior map for GH and SI mice. (**D**) Time spent in Regions 9 and 10 were significantly increased in SI mice compared to GH controls. Other regions of behaviors had no differences between the groups. (**D**) Multi-logistic regression was performed on all behaviors identified by the Motion Mapper pipeline. Feature importance is shown with logistic regression coefficients. (**E**) Nose to nose distance was not significantly different between GH and SI mice. (**F**) Raster plots from a single animal in each group, depicting manually scored behavior and nose-nose distance during SAUSI. (n=18; independent samples t-tests).

Thirty-six high speed videos were loaded into Social LEAP Estimates Animal Poses (SLEAP), which allows for the identification and pose estimation of multiple behaving mice (Pereira et al., 2022). We tracked 8 body points on each mouse using SLEAP (Fig. 2a) and cleaned the data to include only 2 tracks. After initial tracking was performed, blind experimenters manually corrected any switched tracks, and interpolated the videos. Next, we extracted pose estimation data and tested whether computationally extracted features could distinguish the “state” of the animal (SI vs. GH conditions). To do so, we used Motion Mapper (Berman et al., 2014), which enables the visualization of changes in feature space identified by stereotyped patterns of motion (Fig. 2a). Using the Motion Mapper pipeline, x/y coordinates for each body point extracted from SLEAP were processed using a series of dimensionality reductions. The raw data was converted into angles, normalized, and analyzed using principal component analyses (PCA) for the first major dimensionality reduction. Next, we used UMAP to compute distances in high-dimensional space and neighboring points and embedded them closely on a new low-dimensional space, allowing for visualization of patterns of motion. Finally, we used watershed segmentation to separate the continuous stereotyped movements into groups (or regions), which were identified as collections of features (Fig. 2a). Motion Mapper identified 10 distinct regions using data from all groups combined (Fig. 2b). Visualization of occupied feature space for each group revealed distinctions between group housed and isolated mice (Fig. 2c). We next evaluated how much time each mouse spent engaging in specific regions and found that there were significant differences between GH and SI mice in regions 9 and 10 (Fig. 2d). Next, to test whether the housing condition of mice could be decoded based on motion-mapper generated features, we employed multi-logistic regression analyses using regions 3–10 to determine housing condition accuracy and importance of each feature (Fig. 2e). Regions 1 and 2 were excluded from analysis because they represent outlier/missing tracked points from SLEAP. This provided a 75% training accuracy, with regions 10 and 9 identified as the most impactful in distinguishing between GH and SI mice. This demonstrates that the state of the mouse (GH or SI) can be mathematically predicted from unbiased, unsupervised feature analysis based on postural tracking data. Finally, using SLEAP tracking, we assessed distance between the nose of the experimental and conspecific mice and found a numerical increase for isolated mice over the course of the test phase (Fig. 2f). When assessing how behaviors change in congruence with nose-nose distance, we found a stark contrast between isolated mice and controls. Isolated mice display more social hesitancy behaviors prior to engaging in social interaction (latency to approach, longer time sheltering in tunnel on the first approach, reversing in the tunnel) and as mice gain proximity, the behaviors switch to social fear – highlighted by the presence of social freezing behaviors (Fig. 2g). In sum, the unbiased identification of stereotyped motion by unsupervised machine learning approaches revealed distinguishable differences between GH and SI mice. Combining this with supervised behavioral analysis techniques revealed that these differences are likely due to changes in the state of the animal, including an increase in social anxiety in isolated mice.

### Isolated males show decreased prosocial motivation and increased aggression in SAUSI.

**Figure 3.**
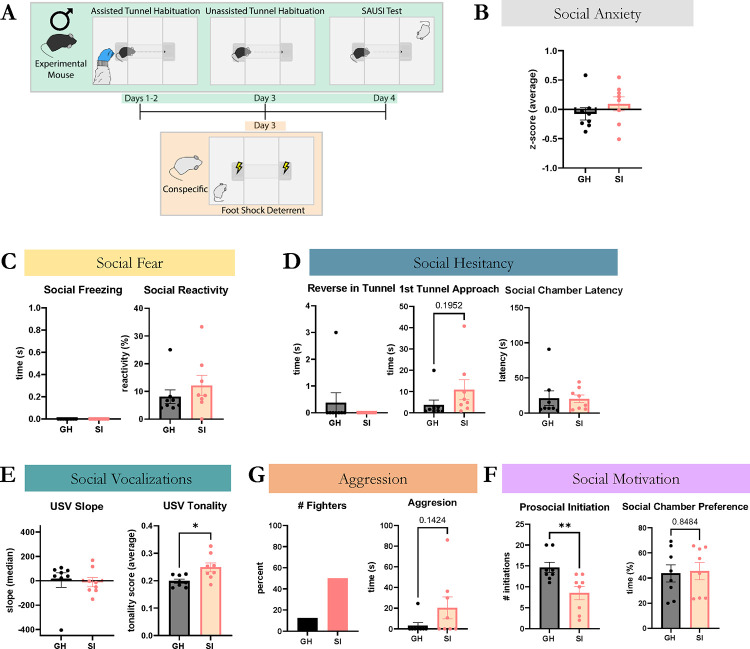
Isolated males display altered social behaviors during SAUSI. (**A**) Timeline for the 4-day SAUSI protocol. (**B**) Males do not show a significant difference in social anxiety between GH and SI mice. (**C**) Isolated males do not display social fear behaviors such as social freezing, and have limited numerical increase in social reactivity. (**D**) Males do not show social hesitancy behaviors. (**E**) Isolated males do not have reduced USV slope, but the tonality of their USVs differs from GH males. (**F**) A higher percentage of isolated males were aggressive compared to GH males. Aggression time was numerically higher (non-significant) in the isolated males compared to the GH males. (**G**) Isolated males had fewer prosocial initiations than GH controls. No differences in social chamber preference between groups. (n=8 ; independent samples t-tests).

To evaluate whether male mice similarly display increases in social anxiety-like behavior following isolation, we tested males using SAUSI (Fig. 3a). Parameters where identical to those used for females with the exception that a slightly larger tunnel was employed to accommodate the larger body size of males (Fig. 1b). Surprisingly, we found that isolated males did not show significant increases in social anxiety-like behaviors when compared to GH males (Fig. 3b). While a subset of isolated mice did display a positive social anxiety score, these mice lacked social fear behaviors – key influencers of social anxiety-like behavior in female mice. Indeed, social freezing was not observed in any of the male mice, and only muted, insignificant increases in social reactivity were found (Fig. 3c). Analysis of USVs revealed changes in the tonality of male calls, with no other significant differences observed (Fig. 3d). Finally, no significant changes were found in social hesitancy behaviors (Fig. 3e). Altogether, this indicates that social isolation in males does not induce social anxiety states, but may manifest in other forms of behavior instead. For example, a higher percentage of SI male mice (50%) engage in aggressive behaviors compared to GH males (12.5%), with the total time spent in aggressive behaviors approaching significance (Fig. 3f). We also found a significant reduction in prosocial initiation, indicating a lack of motivation for positive social interactions (Fig. 3g). These are consistent with prior studies demonstrating that prolonged social isolation results in reduced pro-social behavior and increased aggression in male mice (Grammer & Zelikowsky, 2022; Koike et al., 2009; Padilla-Coreano et al., 2022; Tan et al., 2021; Zelikowsky et al., 2018a). These data indicate that chronic social isolation promotes antisocial behaviors in male mice, but social anxiety-like behavior in females.

### SAUSI uniquely reveals social anxiety-like behavior.

**Figure 4.**
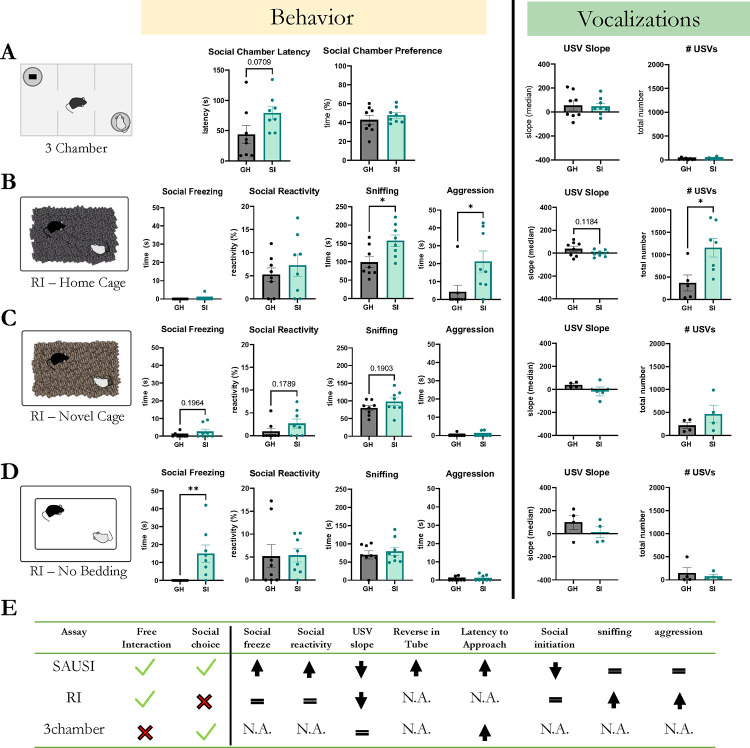
Social anxiety during SAUSI compared to traditional behavioral assays. (**A**) During the three-chamber test, isolated mice showed a trend for increased latency to approach the social chamber. There was no difference in social chamber preference or in vocalizations. Vocalizations were fewer than in other tasks but are unaltered by isolation. (**B**) During home cage resident intruder (RI), isolated mice did not display social fear behaviors, but did engage in higher amounts of sniffing and aggression. In isolated mice, the slope of USVs tended to be lower, while they significantly made more USVs overall. (**C**) During RI-novel cage, no significant differences in behavior or USVs were found. However, small amounts of social fear behaviors were present in isolated mice. (**D**) During RI-no bedding, isolated mice displayed social freezing behavior and minimal aggression. The slope of USVs tended to be lower in isolated mice across all conditions, but did not always reach significance. (**E**) Summary table of the advantages of SAUSI over other assays and how behaviors differ across each test. (independent samples t-tests). *NA, not applicable.*

To test whether SAUSI is unique in its ability to allow for the assessment of social anxiety-like behavior or whether social anxiety could be comprehensively probed using other, well-established behavior assays, we compared behavior on SAUSI to both behavior in the 3-chamber social interaction assay (Nadler et al., 2004; Yang et al., 2011b) and the Resident Intruder Assay (Kemble, 1993; Koolhaas et al., 2013a). We performed a classic 3-chamber social interaction assay (Nadler et al., 2004; Yang et al., 2011b). Briefly, experimental mice were placed in the center chamber of a 3-chamber arena. A sex-and age-matched conspecific was placed in one chamber under an overturned pencil cup, while an inanimate object (e.g. plastic block) was placed under a pencil cup in the opposite chamber. Given limited social interaction due to the restrictive nature of the 3-chamber design, we are unable to assess fear specific behaviors during this assay. However, we were able to discern one aspect of social hesitancy in isolated mice – the increased latency to enter the social chamber (Fig. 4a). Likewise, there was no difference in social preference in the 3-chamber assay (Fig. 4a), similar to what was observed in SAUSI (Figure 1 – figure supplement 3a). In addition, mice vocalized in the 3-chamber (Fig. 4a), but with far fewer USVs observed than in SAUSI (Figure 1 – figure supplement 3c). There were also no significant differences in call slope or other call features, in contrast to effects observed using SAUSI (Fig. 4a). This data reveals the importance of the free interaction featured in SAUSI, in that while we were able to measure one aspect of social hesitancy, all other social fear-related behaviors were largely absent. Furthermore, limiting social interaction with physical restriction altered the number and quality of USVs in mice. These differences in social communication could impact the behavioral dynamics between two mice, further distancing them from a naturalistic social setting (Keesom et al., 2017; Premoli et al., 2020; Sangiamo et al., 2020; Zhao et al., 2021).

Next, to determine if we could recapitulate the effects of SAUSI by turning to a free social interaction assay, we tested mice in the Resident Intruder (RI) assay – another widely-used behavior assay which traditionally takes place in the home cage of the experimental mouse (Kemble, 1993; Koolhaas et al., 2013b). A BALB/c conspecific (“intruder”) was placed in the experimental mouse’s home cage (GH vs. SI). Experimental mice were each tested with a 3-minute baseline (no intruder) and 10-minute test phase (+intruder). In contrast to results obtained using SAUSI, we found a lack of social fear behaviors (Fig. 4b). Instead, we found a significant increase in sniffing and aggression following SI (Fig. 4b). There was also a significant increase in the number of USVs produced by isolated mice, however changes to slope were not significant (Fig. 4b). We hypothesized that the failure to find any SI-induced changes in social fear behaviors may be due to competition with territorial behaviors (sniffing and aggression) (Mongeau et al., 2003), as well as the inescapability of the environment (Weber et al., 2022).

To determine whether territorial behavior competes with anxiety behavior to eliminate social fear in a RI assay, we next performed RI in a novel cage (with new bedding), as RI-aggression has been shown to be highly context-dependent (Koolhaas et al., 2013b; Weber et al., 2022). We found that in a novel cage environment, isolation did not elicit distinguishable features that were observed in other assays (Fig. 4c,e). Indeed, while social freezing and aggression were minutely present in SI mice during novel cage testing (Fig. 4c), the magnitude was smaller than observed in other assays (Fig. 1c, 4a, 4d). To test whether this could be due to the presence of bedding material, as opposed to plastic flooring (used in SAUSI), we tested animals on the RI assay in a novel cage without bedding. RI-novel-no bedding revealed significant increases in social freezing behavior (Fig. 4d). This indicates that the absence of bedding is a driving force of social freezing.

## DISCUSSION

Here we introduce SAUSI, a novel assay which can be used to assess social motivation, hesitancy, decision making, and in depth free social interaction in mice. SAUSI achieves this goal by enabling mice to choose between a “social” chamber and a “home” chamber using a tunnel for decisive access to either side, and combines this element of social choice and motivation with the ability to freely interact with a conspecific on the social side. Using this novel design, we have been able to wholistically characterize social anxiety behavior, identifying critical features which drive this state: social fear, vocal communication, social hesitancy, social motivation and prosocial interaction behaviors. SAUSI also enables us to assess generalized anxiety through non-social freezing and thigmotaxis behavior during baseline. Machine learning and computational approaches further revealed unbiased differences in the stereotyped movements of socially isolated versus group-housed mice during SAUSI. Pairing this with the supervised behavioral analysis revealed the impact of isolation to induce impactful changes in behavior including the induction of social anxiety states.

One intriguing discovery we made is that SAUSI allows for a number of behaviors to be displayed as a series along a continuum. Interestingly, this continuum seems largely governed by the imminence of the conspecific, consistent with theories which posit that animals will display a series of stereotypical and species-specific defense behaviors across a threat imminence continuum (Abend, 2023; Fanselow, 2022; Mobbs et al., 2020; Perusini & Fanselow, 2015). Indeed, when the distance between the experimental mouse and the conspecific mouse (or social “threat”) is large, animals display social avoidance behaviors such as increased latency to approach the social chamber. These can be thought of as examples of “pre-encounter” behavior, as mice are in a position to avoid potentially threatening social contact. As experimental mice gain proximity to the conspecific, but prior to contact, behavior continues to progress on a “social threat” imminence continuum. This can be indexed by increased hesitation to approach the social chamber, sheltering for long periods in the tunnel, and/or reversing out of the tunnel after the decision has been made to cross over to the social chamber. These behaviors may indicate an ongoing assessment of threat and incorporate retreat as the conspecific is closer and closer. Finally, once mice enter the social chamber and begin to interact with the conspecific, encounter & circa-strike behaviors can be identified. These include social freezing and heightened social reactivity in response to being investigated. This continuum elucidated by SAUSI reveals the emergence of a state of social anxiety, which can largely be indexed by early “pre-social encounter” social behaviors, consistent with the view that anxiety more generally is a state which is characterized by pre-predator encounter behaviors (Abend, 2023; Fanselow, 2022; Mobbs et al., 2020; Perusini & Fanselow, 2015).

In the current study, behavioral changes induced by social isolation were found to be sex dependent. While isolated females displayed a myriad of social anxiety-like behaviors, isolated males did not show social anxiety. Instead, isolated males had a reduction in social initiation and an increase in aggression, indicating more of an anti-social rather than anxious emotional state. The presence of stress-induced social anxiety in female but not male mice is consistent with human literature. Women have been found to be impacted by stress and social anxiety at higher rates than men (Asher et al., 2017; Asher & Aderka, 2018). Some evidence suggests that social anxiety has altered clinical representation based on sex (Asher & Aderka, 2018; Barnett et al., 2021; Caballo et al., 2014). Furthermore, while chronic social stress has been linked to the development of social anxiety (Richey et al., 2019), the different impacts that social isolation has on social anxiety in men vs. women is unclear. This evidence suggests the importance of studying both male and female subjects in social anxiety research, and highlights the interesting possibility that diverging states can emerge from the same stressor (Chaplin et al., 2008; Goldfarb et al., 2019; Tan et al., 2021). These differing outcomes provide additional motivation to understand the neurobiology of isolation and the manifestation of distinct behaviors that arise from this state.

Our results demonstrate that isolation-induced changes in behavior are largely assay- and context-dependent. Certain behavioral assays or environmental contexts reveal territorial-driven changes to behavior following SI, while others are more sensitive to social motivation changes. Our results indicate that it is critical to choose an assay that accurately allows you to test your hypothesis, otherwise, competing behaviors may obscure findings. SAUSI allows for the comprehensive assessment of social anxiety behaviors – in contrast, assays with bedding shift behavior towards territorial behavior. Our findings suggest that bedding may also offer a source of familiarity and comfort to the mice that mitigates anxiety responses, or could offer an alternative anxious response to being investigated, such as digging instead of freezing. Other assays such as the 3-chamber lacks free behavior interaction, and a home cage testing removes the element of choice. Ultimately, this reveals SAUSI as a unique assay that is particularly fit to examine behaviors related to social anxiety.

While we expected isolation to induce aggression in our RI assay (Grammer & Zelikowsky, 2022; Koike et al., 2009; Tan et al., 2021; Zelikowsky et al., 2018b), we were surprised to find SI-induced aggression in SAUSI for both males and females. Two roles emerge in aggressive interactions - offensive and defensive. These have been described extensively (D. C. Blanchard & Blanchard, 1990; R. J. Blanchard et al., 2006; R. J. Blanchard & Blanchard, 1989; Koolhaas et al., 2013a) and are indicated primarily by posture, advancement, and location of attack or submissive posture and flight behaviors, respectively. However, the circumstances and emotional states which motivate attack are less often discussed. Recent studies suggest that there may be a rewarding component for the aggressor which could provide motivation to initiate aggression (proactive rather than reactive aggression) (Couppis & Kennedy, 2008; Falkner et al., 2016; Mondoloni et al., 2022; Padilla-Coreano et al., 2022). Other theories suggest the establishment of dominance (Koolhaas et al., 2013a). The SAUSI setup reveals that this isolation induced aggression is likely intrinsically motivated (proactive) in nature (Couppis & Kennedy, 2008; Falkner et al., 2016; Mondoloni et al., 2022; Padilla-Coreano et al., 2022). This is demonstrated by isolated mice repeatedly choosing to enter the social chamber in order to engage in immediate and repeated attack or mounting without provocation or reciprocation. Additionally, if aggression were reactive in nature, we would have expected to find similar rates of aggression towards a conspecific across all tests, especially when forced to interact with no escape options from the cage. However, we failed to see aggression in either the novel cage or no-bedding condition. We believe that the stark differences in aggression across contexts of forced interaction indicate that this is proactive behavior instead. Our findings suggest that the motivation to be aggressive is intrinsically heightened for isolated mice, increasing the initiation of aggression altercations with a conspecific, unprovoked. Further testing is needed to assess the role of dominance in influencing the motivation for aggression (Koolhaas et al., 2013a). Additionally, studies that investigate aggression seeking behavior rely on learned reward tasks such as operant aggression seeking or aggression conditioned place preference, which require training and learning on behalf of the experimental mice (Aleyasin et al., 2018; Falkner et al., 2016). SAUSI provides the ability to assess motivation for aggression in a more naturalistic setting, without training, and with free interaction.

Social anxiety, the fear of social situations, affects approximately 25 million adults in the United States alone (Rich, 2023; Social Anxiety Disorder, n.d.). Each year anxiety disorders have steadily risen (Goodwin et al., 2020), particularly in response to the growing epidemic of loneliness (Weissbourd et al., 2021), suggesting that far more people are currently impacted. Despite the prevalence of social anxiety, our understanding of its etiology and underlying neurobiology is limited, hindered by the lack of animal models for social anxiety behavior. Our findings impact the field of social anxiety research by providing an in-depth mouse model for social fear and social anxiety studies in the future. This provides a foundation for understanding the biological mechanisms that are altered by social isolation and which lead to social anxiety states and the behaviors that are produced by these states.

## MATERIALS AND METHODS

### Data code and availability

The SAUSI apparatus is open-source, and design files are freely available online at: https://github.com/ZelikowskyLab/SAUSI_paper. In addition, we have made all analysis code for the figures in this paper available at https://github.com/ZelikowskyLab/SAUSI_paper.

### Subjects

One hundred C57Bl/6 mice and eighty BALB/c mice were housed in cages (Thoren type 9 https://thoren.com/?page_id=71) with Teklad pelleted bedding (https://www.inotivco.com/7084-pelleted-paper-contact-bedding) on a ventilated rack in a 12 hr light/dark cycle with full time access to food (Teklad Global Soy Protein-Free Extruded Rodent Diet 2920X) and water. All animal work was reviewed for compliance and approved by the Institutional Animal Care and Use Committee of The University of Utah (IACUC protocol #00001504).

### Design and construction of SAUSI

The SAUSI apparatus was designed in CAD and built by the machine shop at the University of Utah. The floors were made of white High Density PolyEthylene (HDPE) plastic, the tunnel (tube) and walls were made of transparent acrylic plastic. The tunnel block (5.3 × 2.5 × 6.3 cm) was made of metal (673 g - heavy material that mice cannot move), and the U-channel tunnel block was 3D printed. Transparent, plastic wall extenders (33 cm tall) were fitted to the exterior walls of the sausi box and secured with paper clips to ensure that mice could not jump out of the apparatus.

Foot shock boards were made by hand using metallic tape (optionally on a nonconductive backing (such as scotch tape) for portability). These were connected via alligator clip wires to the floor of the Med Associates fear chamber boxes. During the conspecific tunnel deterrent session, the footshock boards are wired directly to the fear chambers for automated shocking. During the SAUSI test day, this connection is mediated by off-on-off buttons for manual shock administration.

Black out curtains were hung surrounding the setup to prevent side preferences due to differences in visual stimuli across the room. They also improve the even distribution of lighting and increase sound dampening. Scent-free anti-static spray was sprayed on the footshock boards and curtains at the beginning of each day in the SAUSI protocol. This reduced the chances of accidental shock due to static.

### Room conditions

A bar was hung above the apparatus for mounting two yellow light lamps facing away from the mice and the single top view camera. Two side view cameras were also mounted to each side of the SAUSI box with permanent fixtures for consistency. The Avisoft microphone was clamped to the exterior middle chamber wall (Fig. 1a–b).

Lighting: 2 lamps facing upward, yellow hue, lux = 22–23 per chamber.

Temperature was 70–75 F in behavior suite, with air conditioning on (which provided low levels of background noise).

Handling with plastic beakers (4” diameter, 5” tall).

On the first day of the SAUSI protocol, mice were weighed. Each experimental mouse was paired with a lighter-weight BALB/c (2 weeks younger) that was sex matched. Each BALB/c was tested with both a GH and SI mouse in counterbalanced order. The hind region of BALB/c mice were colored with Stoelting animal markers (https://stoeltingco.com/Neuroscience/Animal-Markers~9769) to be identified throughout the training and testing.

### Mice and isolation

All mice were purchased from Charles River Laboratories. The strain of experimental mice were C57 BL/6, whereas the strain of conspecific were BALB/c mice. All experimental mice arrived at the University of Utah vivarium facilities at 5 to 6 weeks old, with three to four mice per cage. All mice were housed on a ventilated rack with dark, opaque plastic dividers stationed in between cages. These features were present reduce the visual, auditory, and olfactory stimuli of surrounding cages. For each experiment, half of the mice were placed in social isolation, while the other half remained in their original group-housed cages. Isolation began at 6 weeks old and lasted for a total of 4 weeks (tested at 10 weeks old). Conspecifics were group-housed with 4 mice per cage and were tested at 8 weeks old (2 weeks younger than experimental mice).

### SAUSI protocol

The SAUSI apparatus contains two outer rooms, one empty and one containing a conspecific. These rooms are connected by a narrow tunnel with a miniature foot shock board on either side, which deter conspecifics from crossing over, and allows for observable decision making in the experimental mice.

To habituate mice to the apparatus, tunnel, and handling, experimental mice are placed in the arena for 5 minutes each on days 1 and 2. During this assisted habituation, experimenters gently guide the mouse through the tunnel every 30 seconds. Every 15 seconds, the experimenter places a hand in the opposite chamber of the mouse to habituate it to movement. This indicates to the mice that experimenters can move and intervene without attempting to handle the mouse every time and thus reduces intervention stress. Time restarts after each time the mouse crosses through the tunnel. For the very first entrance on the first day, mice are held by the tail with all 4 paws on the ground with their face directed near the entrance of the tunnel. From there, experimenters patiently wait until mouse enters the tunnel. Once all four paws are in, the mouse is blocked from reversing out of the tunnel (Fan et al., 2019). After the first entrance, “corralling” the mouse close to the tunnel entrance without touching it is sufficient for them to enter the tunnel and reduces stress. On the third day, mice are placed in the apparatus and monitored to ensure the mice use the tunnel at will (inspired by hierarchy tube habituation – fan et al). The conspecifics undergo a 5-minute foot shock deterrent session on day 3 to prevent them from crossing to the other side and blocking the tunnel entryway.

On day 4, the social behavior test consists of a 3-minute habituation period for the experimental mouse followed by a 10-minute test phase with the conspecific present (figure 1b). To begin the baseline phase, the tunnel is blocked off in the starting chamber. The experimental mouse is placed in one chamber (alternating between left and right for each trial) and the block is removed to signify the start of the 3 minutes. Once three minutes has past, the experimenter traps the mouse on the side it originally started on using the tunnel block. The conspecific is placed in the opposite chamber and the block is removed to signify the start of the 10-minute test phase. During the social test, the foot shock boards are controlled manually by a button and can be used if a conspecific steps on the foot shock board while the experimental mouse is not also on it. This only happens occasionally and can be reduced by minimizing the number of times each conspecific is used. To transport mice to and from the SAUSI apparatus, the tunnel is first blocked off and a plastic beaker (4” diameter, 5” tall) is used to transition the mouse back to its home cage.

### 3-Chamber

Lighting, video, and audio recording (as well as all other factors: curtains, wall extenders, anti-static spray, cup handling) were set up identically to SAUSI. The temperature was 70–75 F in the behavior suite, with air conditioning on (which provided low levels of background noise). The apparatus used for SAUSI was also used for 3-chamber testing, with the tunnel replaced by removable doors and with no foot shock boards present. Cups used to separate conspecifics (https://www.noldus.com/applications/sociability-cage) were placed in the top left or bottom right of the respective chamber and weighted down by water bottles. Videos were recorded from the overhead camera using MediaRecorder and scored using automated tracking in EthoVision. USVs were captured using Avisoft and were analyzed with DeepSqueak.

The 3-chamber task began with a 5-minute baseline phase, where experimental mice were placed in the middle chamber with nothing else present in the apparatus. The baseline phase began once the experimenter removed the doors, allowing the mouse to explore all 3 empty chambers. After the baseline phase, mice were then ushered back into the middle chamber, with access to other chambers blocked by doors in order to transition to the test phase. The test phase lasted 10 minutes and consisted of an inanimate object (black plastic block) under a cup in one chamber, and a conspecific mouse under a cup in the opposite chamber.

The “social” side was counterbalanced for each trial. Each conspecific was paired with a GH and an SI mouse during the test day, counterbalanced for order. The apparatus, cups, water bottles, and doors were cleaned with 70% ethanol between each trial.

For conspecifics, BALB/c mice that were sex matched and 2 weeks younger than experimental mice were used.

### Resident Intruder

Lighting was provided by two lamps facing upward with yellow hue, lux = 14–15. Temperature was 70–75 F in behavior the suite, with air conditioning on (which provided low levels of background noise). Videos were recorded from an overhead camera (43 cm from the cage floor) using MediaRecorder and manually scored in EthoVision. USVs were captured using Avisoft (microphone 38.1 cm from the cage floor) and were analyzed with DeepSqueak. A box surrounded each cage during testing and contained a ledge to prevent mice from exiting the cage during testing (https://github.com/ZelikowskyLab/SAUSI_paper).

The RI tests began with a 3-minute baseline phase, where the experimental mouse was observed in the cage on its own. This was followed by a 10-minute test phase, in which a conspecific mouse was placed in the cage with the experimental mouse.

Cages used (Thoren type 9- https://thoren.com/?page_id=71) were 19.56 × 30.91 × 13.34 (cm.) with a floor area of 435.7 (Sq. cm.). Bedding that mice were continuously housed in (Teklad pelleted bedding - https://www.inotivco.com/7084-pelleted-paper-contact-bedding) or new bedding of the same type were used for RI-home cage and RI-novel bedding. Nestlets were removed for visibility throughout the task. Food and water were not accessible during the baseline and test phases of the task.

Group-housed mice that were not actively being tested were placed together in a clean holding cage. Each group-housed mouse shared an “untested” holding cage with their cagemates prior to testing. After being tested, they were placed in another clean “tested” holding cage so that no untested mice were exposed to tested mice.

For conspecifics, BALB/c mice that were sex matched and 2 weeks younger than experimental mice were used. Each conspecific was paired with a GH and an SI mouse during the test day, counterbalanced for order.

### Behavior and Vocalization Analysis

**Table:** 

Behavior	Event Type	Description	equation
Face Sniff	Start-stop	Sniff in front of the ears	
Body Sniff	Start-stop	Sniff behind ears (including ears) and anterior to anogenital/tail regions	
Anogenital Sniff	Start-stop	Sniff anogenital region (below the tail)	
Tail Sniff	Start-stop	Sniff tail only	
All Sniff	Start-stop	All sniffing combined	Allsniff=Face+body+anogenital+tailsniff
Conspecific sniff*	Point event	Any new sniff bout by the conspecific	
First Tunnel Approach	Start-stop	In tunnel before first time entering the social chamber (can be multiple times before entering). must have all 4 paws in tunnel.	
Reverse in Tunnel	Start-stop	Backing out of the tunnel after all 4 paws entered.	
Social Freeze	Start-stop	freezing in response to being sniffed by the conspecific. Can be during or just after being sniffed by the conspecific (within 1 second).	
Social Reactivity	Point event	experimental mouse reacts when conspecific sniffs it (within 1 second of the end of a sniffing bout from the conspecific). Reacting can include retreating, darting, vigilance, flinching, jumping, or attempting to exit apparatus.	$\frac{social\;reactivity\;bouts}{conspecific\;sniff}\;\text{*}\;100\text{\%}$
Social Initiation	Point event	Any time the c57 approaches the BALB/c to sniff and closes the gap between them/comes from more than one body length distance away. The BALB/c does not close the gap.	
Social Chamber Preference	Start-stop	Preference to spend time in the social chamber.	$\frac{social\;chamber\;time\;\left(s\right)}{total\;time\;\left(s\right)}\;\text{*}\;100\text{\%}$
Groom	Start-stop	Self grooming	
Non-social freeze	Start-stop	Freezing not in response to being sniffed by the conspecific.	
aggression	Start-stop	Attack, biting, mounting	
jump	Point event	Attempt to exit the apparatus. All 4 paws leave the ground.	

* all behaviors are referencing the experimental mouse except for conspecific sniff, which measures the bouts of sniffing performed by the BALB/c mouse.

Videos were recorded in MediaRecorder. The top view perspective was used for automated analysis latency and time spent in each chamber with EthoVision (Noldus et al., 2001) as well as postural data tracking in SLEAP (Pereira et al., 2022). Side view perspectives were used for hand scoring behaviors in EthoVision.

DeepSqueak was used to analyze all audio recording data from avisoft.

SLEAP (Pereira et al., 2022) was the program used for postural tracking. All videos used for tracking were from the top camera view and were trimmed to only include the test phase. These videos were used for training in SLEAP with >600 frames manually labelled. The “export labels package” feature from the SLEAP user interface was used to export labelled frames, which were uploaded in google colab, where training was performed (Google Colab file accessible using the “train on google colab” feature in the SLEAP user interface). Bottom-Up processing was used for training with default settings. For inference, tracker was set to “flow”, clean_instance_count was set to 2, track_window was set to 30, and all remaining settings were left as default. Google Colab was used for training and inference due to high computational needs.

The Motion Mapper pipeline (Berman et al., 2014) was used for unsupervised behavioral analysis. (modified code available here https://github.com/ZelikowskyLab/SAUSI_paper).

Python and matlab were both used to generate nose to nose distance calculations, behavior rastor plots, all and all regression data (code available here https://github.com/ZelikowskyLab/SAUSI_paper).

GraphPad PRISM was used to generate all statistics and graphs comparing GH and SI conditions.

### Exclusion Criteria

1 mouse was excluded from the figure 1d data because the conspecific stepped on the footshock board (and received a shock) during the first tube entrance of the experimental mouse, which can impact latency to approach, first tube approach time, and reversing in tube. Other pre-defined exclusion criteria for SAUSI include the experimental mouse failing to use the tunnel by the 3^rd^ habituation day, receiving an accidental foot shock, or conspecific aggression. None of these criteria were met throughout experimentation.

Predefined exclusion criteria for RI experiments included BALB/c aggression. 4 mice were excluded from figure 4 “# USVs” data due to BALB/c aggression. All other scores include data from these mice only prior to the first bout of BALB/c aggression.

## Supplementary Material

Supplement 1

Supplement 2

## Figures and Tables

**Key resources table:** 

Purpose	Resource	Link or Information
mice	C57BL/6J and BALB/c– Charles River Laboratories	https://emodels.criver.com/search?species=Mouse
cameras	Basler GigE camera acA1300-60gc	https://my.noldus.com/shared/knowledge-base/answer/256
room setup	overhead pole for camera and lighting mount	Fudesy Photo Video Studeo 10Ft Heavy Duty Adjustable Backdrop Stand (Amazon)
room setup	blackout Curtains (Amazon)	Joydeco Blackout Curtains (Amazon)
room setup	anti-static spray	Plant Therapy Unscented Natural Wrinkle Releaser (Amazon)
lighting	gooseneck lamps with clamping base	GLFERA (Amazon)
microphone	avisoft	https://avisoft.com/ultrasound-microphones/cm16-cmpa/#40011
SAUSI box and tunnels	CAD files (machine shop)	https://github.com/ZelikowskyLab/SAUSI_paper
foot shock board supplies	metallic tape	GENNEL 5mm × 20m Conductive Cloth Fabric Adhesive Tape (Amazon)
foot shock board supplies	buttons	Starelo 19mm 3/4” Momentary Push Button Switch (Amazon) – push & hold-ON; release-OFF
foot shock board supplies	alligator clip wires	Electronix Express 2 Wire 30 Ft Retractable Test Leads, 18 Gauge Wire with Alligator Clips (Amazon)
foot shock board supplies	power supply	Med Associates Fear Chamber and Aversive Stimulator/Scrambler https://med-associates.com/product/aversive-stimulator-scrambler-module/
