# Supplementary material for: SAUSI: an integrative assay for measuring social aversion and motivation
Source: bioRxiv. 2024 Dec 7:2024.05.13.594023. Originally published 2024 May 14. Preprint. [Version 2] doi: 10.1101/2024.05.13.594023 (PMC11118329; doi:10.1101/2024.05.13.594023)

Figure 1 – figure supplement 1

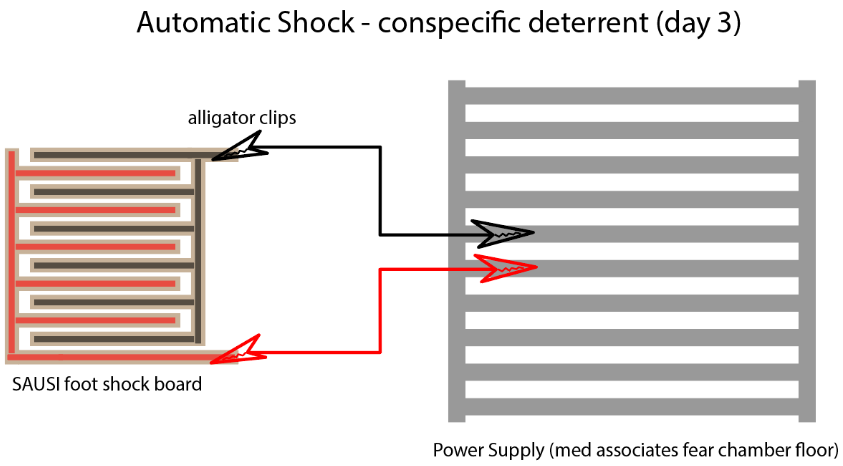

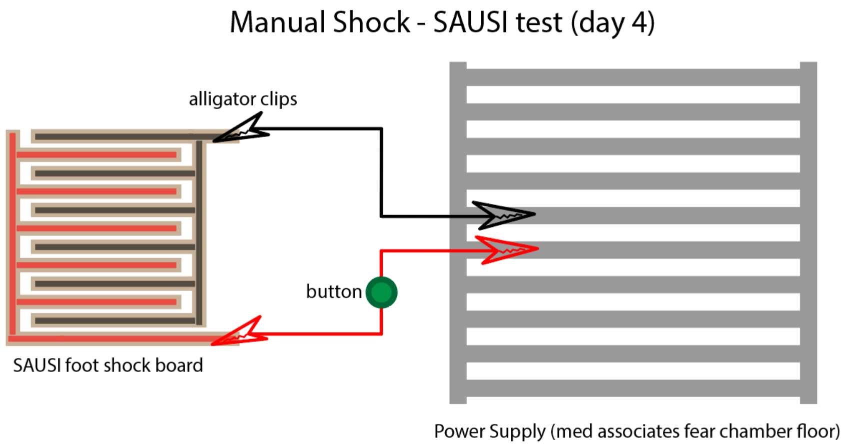

Figure 1 – figure supplement 2

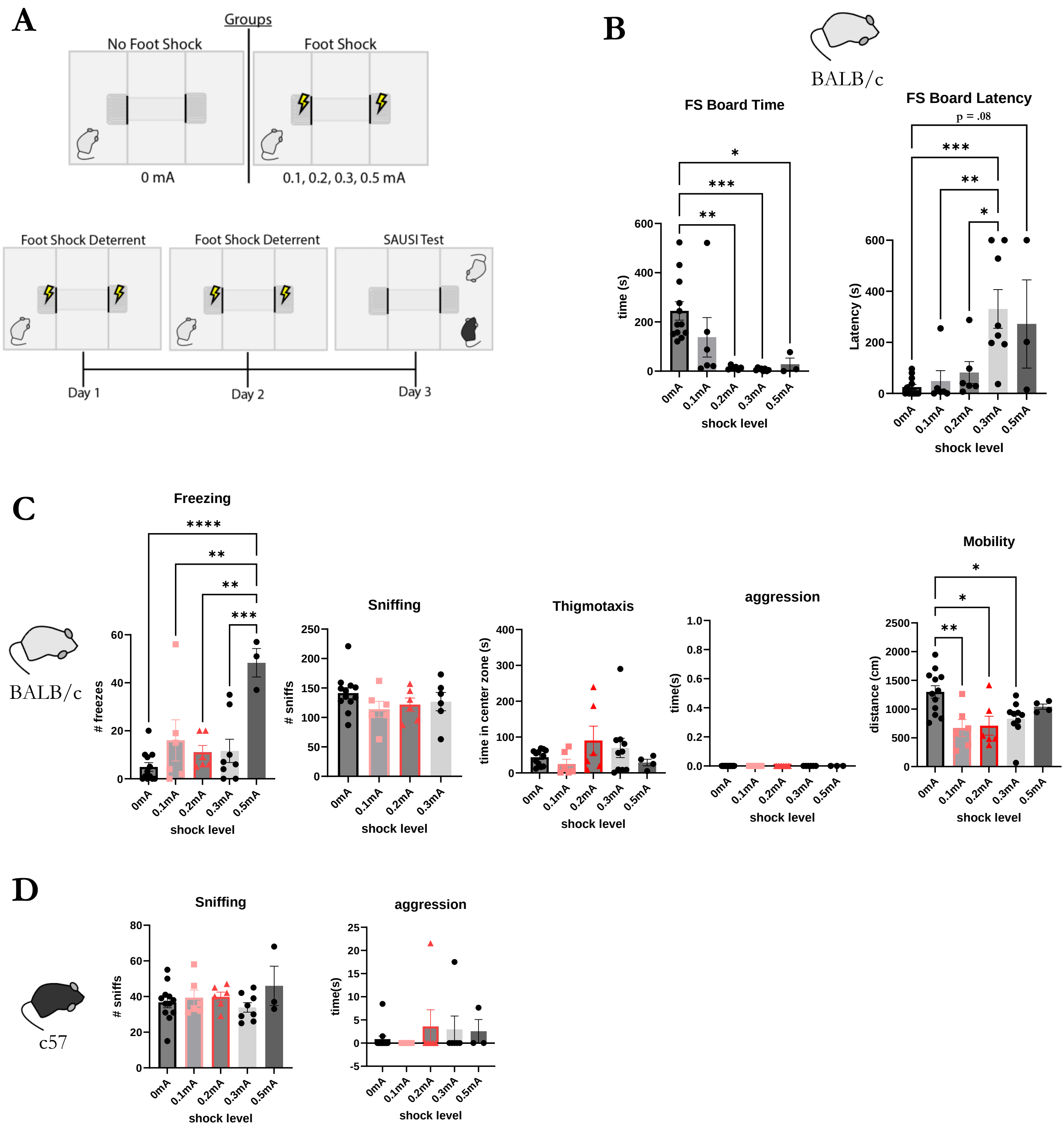

Figure 1 – figure supplement 3

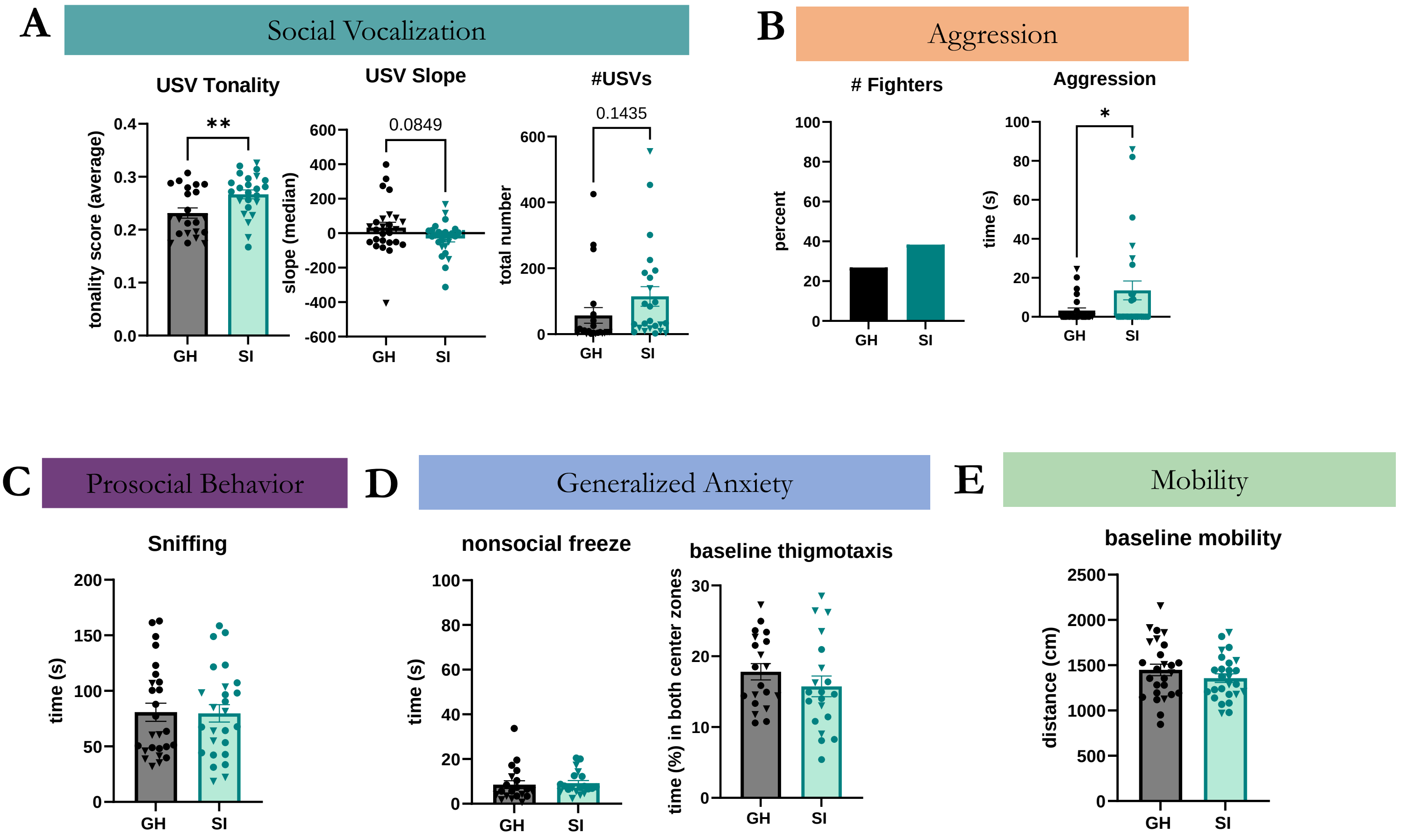

Figure 3 – figure supplement 1

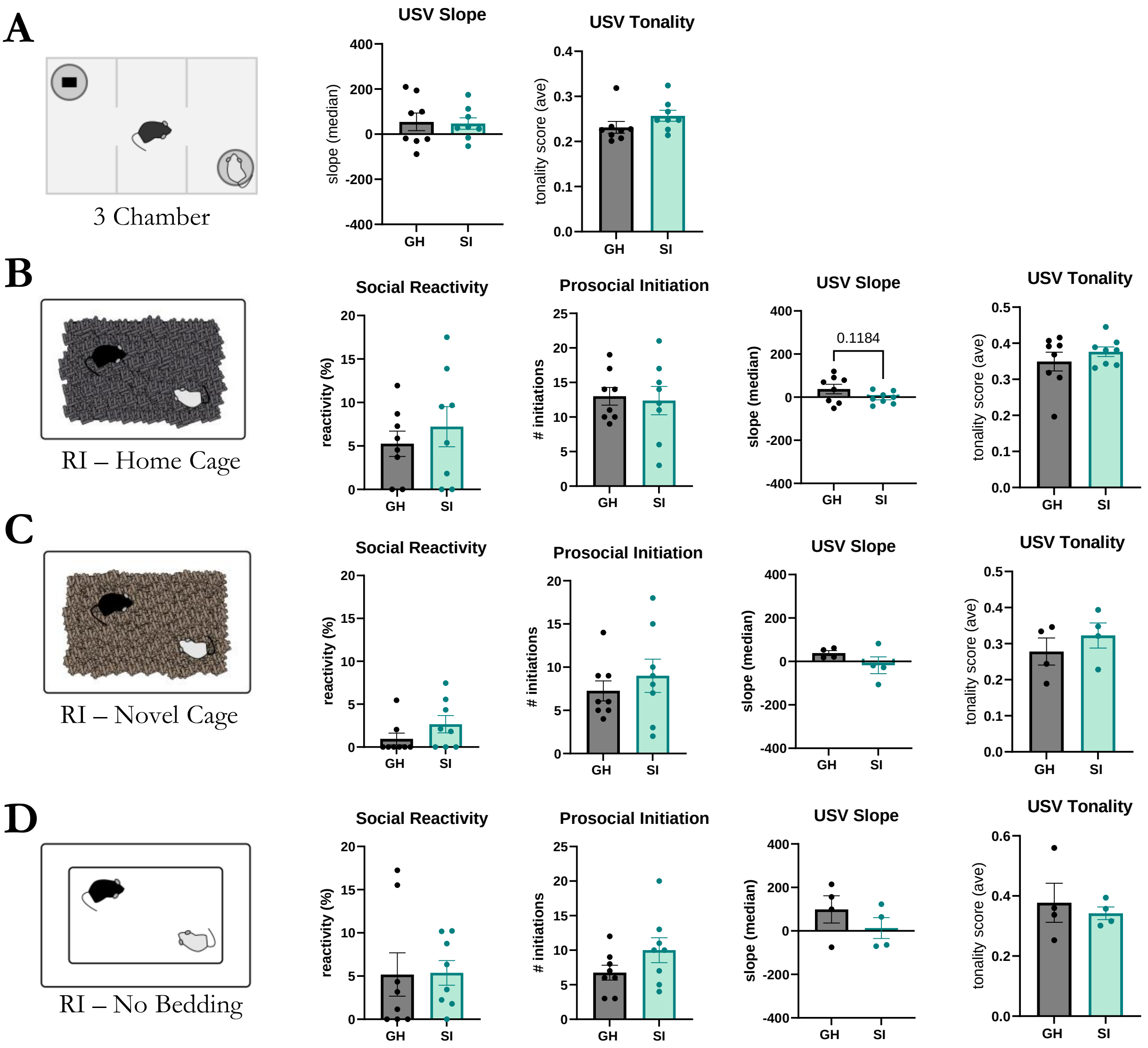

Figure 4 – figure supplement 1

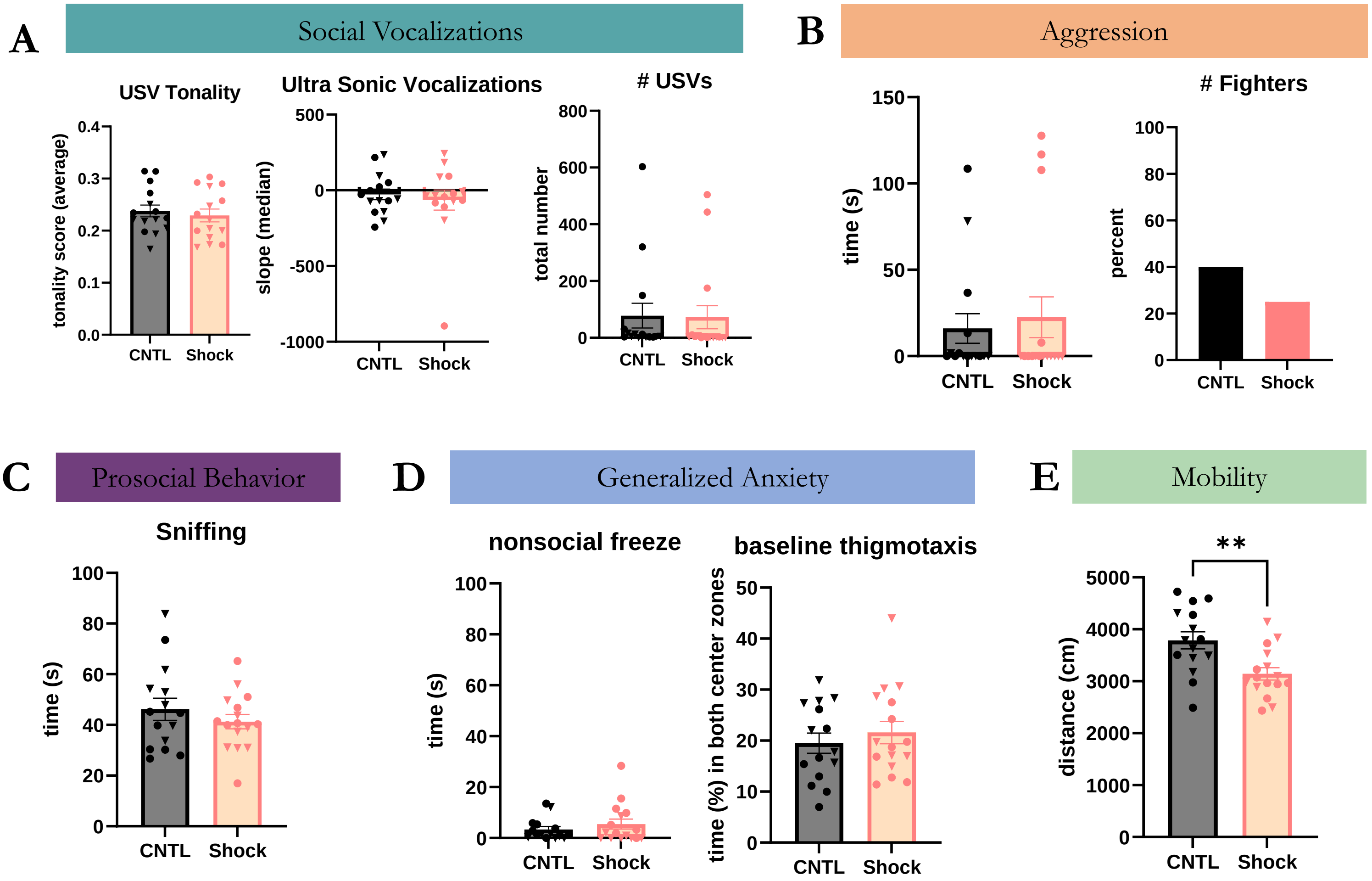

Supplement: Supplement 1 [file media-1.pdf]
