# Supplementary material for: SAUSI: a novel assay for measuring social anxiety and motivation
Source: bioRxiv. 2024 May 14:2024.05.13.594023. Preprint. [Version 1] doi: 10.1101/2024.05.13.594023 (PMC11118329; doi:10.1101/2024.05.13.594023)

Figure 1 – figure supplement 1

Automatic Shock - conspecific deterrent (day 3)

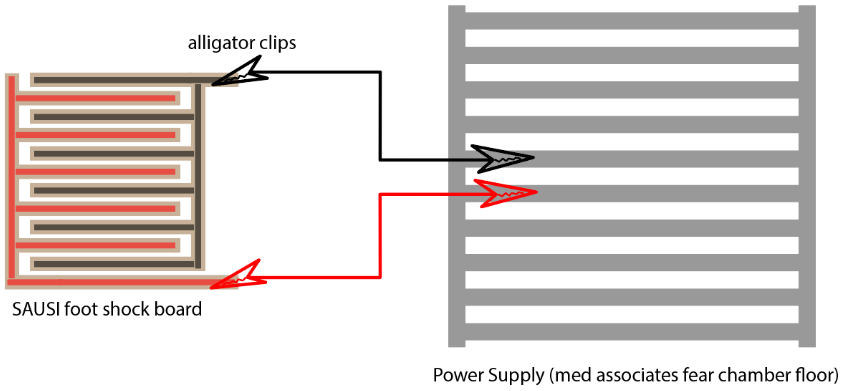

Manual Shock - SAUSI test (day 4)

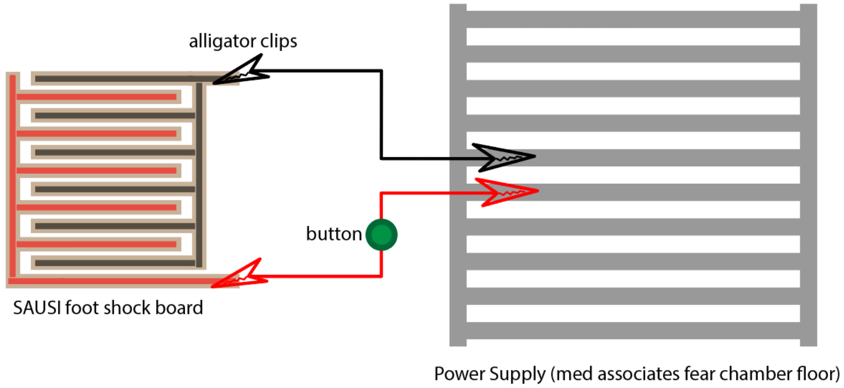

Figure 1 – figure supplement 2

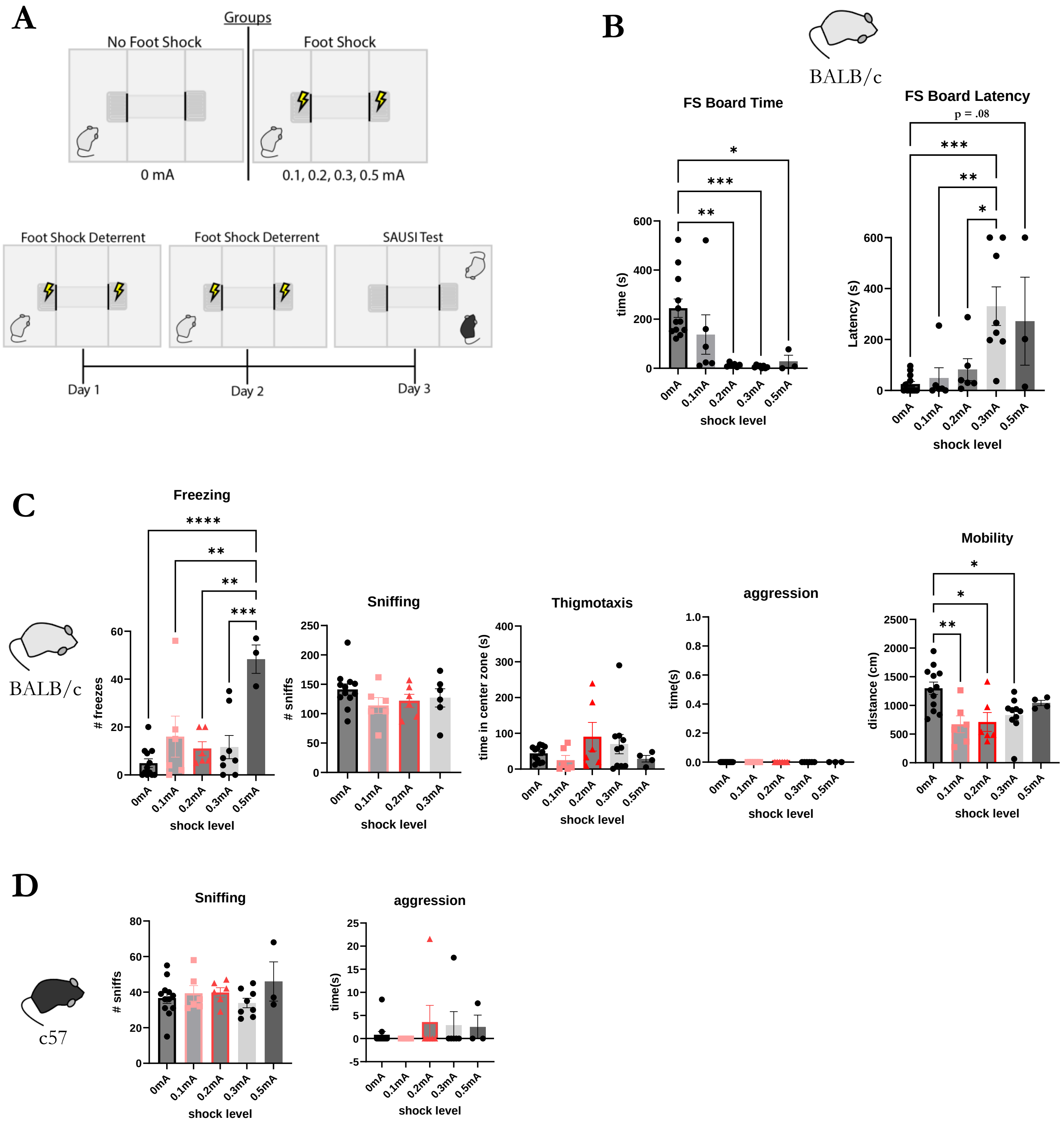

Figure 1 – figure supplement 3

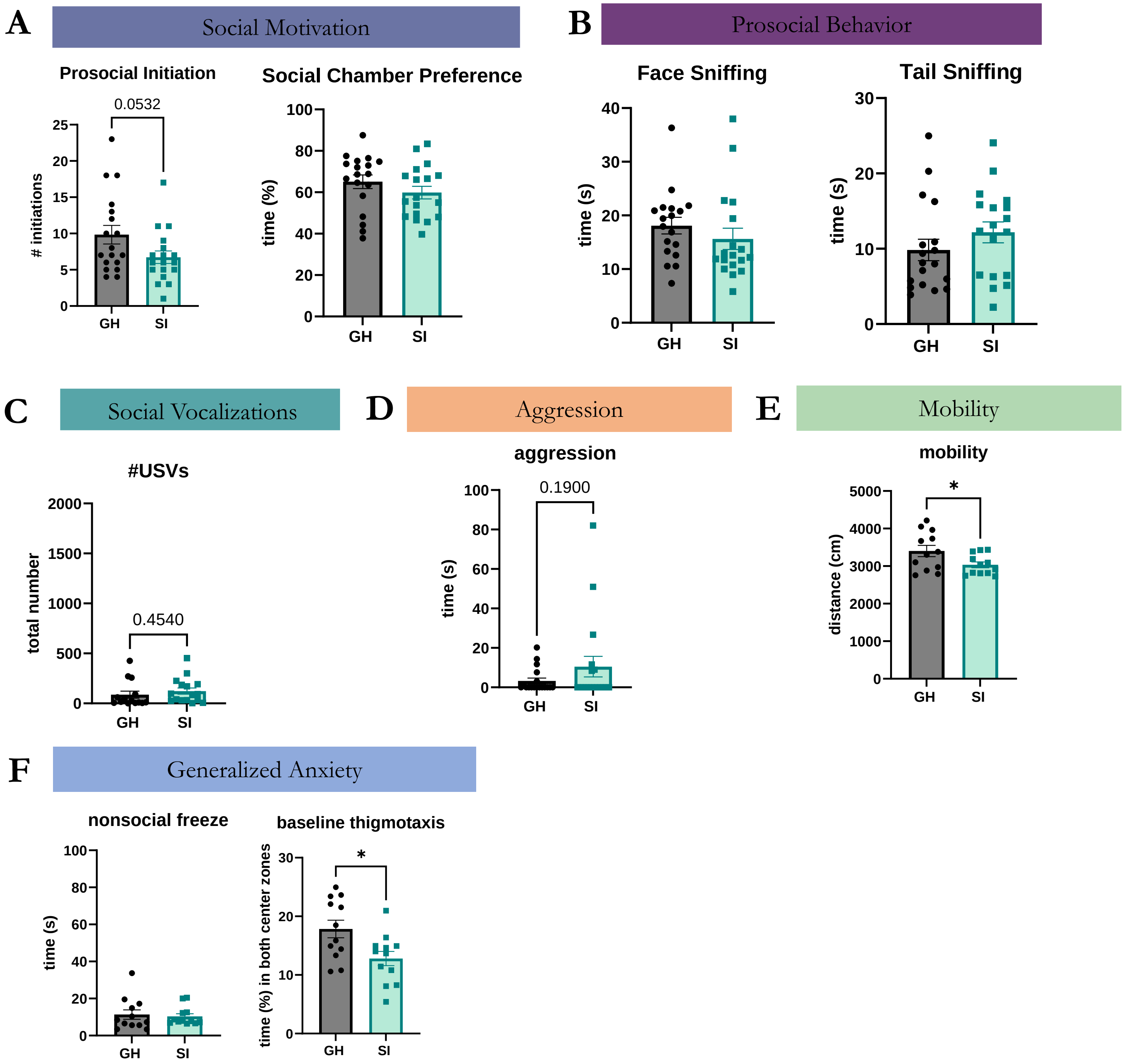

Supplement: Supplementary file 1 [file media-1.pdf]
